# The impact of robot-assisted frameless brain biopsy on performance differences between junior and senior neurosurgeons

**DOI:** 10.1371/journal.pone.0362271

**Published:** 2026-11-05

**Authors:** Carolin Albrecht, Tobias Mahler, Viktor M. Eisenkolb, Benedikt Wiestler, Claire Delbridge, Chiara Negwer, Bernhard Meyer, Arthur Wagner

**Affiliations:** 1 Department of Neurosurgery, Technical University Hospital of Munich, TUM School of Medicine and Health, Munich, Germany; 2 Department of diagnostic and interventional Neuroradiology, Technical University Hospital of Munich, TUM School of Medicine and Health, Munich, Germany; 3 Department of Pathology and Neuropathology, Technical University Hospital of Munich, TUM School of Medicine and Health, Munich, Germany; Weill Cornell Medicine, UNITED STATES OF AMERICA

## Abstract

**Objective:**

Frameless stereotactic brain biopsy is a standardized and routinely performed neurosurgical procedure, frequently carried out by residents under supervision in academic centers. Robot-assisted (RA) guidance has been introduced to standardize workflow and reduce operator-dependent variability. This study investigates whether RA frameless biopsy reduces experience-related performance differences between residents and attending neurosurgeons compared with a manually operated frameless system (MFS).

**Methods:**

We performed a retrospective, non-randomized cohort study of consecutive patients undergoing RA frameless stereotactic brain biopsy for intracranial lesions at a tertiary academic center between 2021 and 2025. Outcomes included perioperative complications, diagnostic yield, and operative time, stratified by surgeon experience level (residents vs. attendings). Results were compared with those of a cohort operated with a MFS.

**Results:**

A total of 98 procedures were analyzed (49 RA, 49 MFS). Across the entire cohort, total operative time was longer with RA than with MFS (115.6 ± 34.1 vs. 97.9 ± 27.2 minutes; p = 0.0036), primarily due to longer setup times. Diagnostic yield and complication rates were comparable between systems. Importantly, subgroup analysis demonstrated that residents using RA achieved operative times, diagnostic yield, and complication rates comparable to attending surgeons. In the MFS cohort, total operative time did not differ significantly between residents and attendings.

**Conclusions:**

RA-assisted biopsy was associated with comparable diagnostic accuracy and safety relative to MFS. Robotic assistance was associated with smaller observed differences in operative efficiency between residents and attending surgeons, suggesting a potential role in bridging experience-related performance differences during neurosurgical training.

## Introduction

Intracranial intraparenchymal lesions often pose a diagnostic challenge, particularly when radiological findings are inconclusive. In some cases, open surgical resection is not the initial therapeutic option either due to the eloquent or deep-seated location, or because alternative treatments may be more appropriate. For instance, patients with primary central nervous system (CNS) lymphoma may respond well to corticosteroid therapy and high-dose methotrexate, making surgical resection unnecessary [1,2].

Stereotactic biopsy remains the gold standard for obtaining histopathological diagnosis. Frameless stereotactic systems, which employ image-guided navigation without the need for a rigid frame, have gained popularity due to improved patient comfort and more flexible intraoperative workflow [3–5]. The Varioguide® system represents a manual frameless system (MFS), enabling real-time tracking of surgical instruments based on preoperative imaging [4].

The integration of robotic assistance into neurosurgical workflows has gained increasing attention in recent years [6,7]. By automating trajectory alignment and reducing operator-dependent variability, robotic systems have the potential to improve the consistency and reproducibility of stereotactic procedures. However, the implementation of robotic guidance also introduces additional workflow steps, including system setup and calibration, which may initially offset potential gains in operative efficiency. Consequently, whether the theoretical benefits of robotic assistance translate into meaningful clinical advantages under routine conditions remains uncertain. Although previous studies have demonstrated improved targeting accuracy and reduced localization error compared with conventional stereotactic techniques [8,9], evidence regarding workflow efficiency, diagnostic performance, and the influence of surgeon experience remains limited.

The Cirq® system (Brainlab, Munich, Germany) is a modular robotic arm (RA) designed to enhance stability and precision during cranial procedures. It provides passive, table-mounted guidance for navigated instruments and is primarily used for drill stabilization and trajectory maintenance in stereotactic interventions [10,11]. The system is designed as an assistive alignment device rather than a fully autonomous robot. While the robotic module facilitates the alignment of the trajectory, the steps of primary setup and more importantly biopsy and needle advancement remain entirely manual. It is fully integrated into Brainlab’s neuronavigation platform, allowing precise trajectory planning based on preoperative imaging.

In academic neurosurgical centers, stereotactic brain biopsies are frequently performed by residents under the supervision of attending surgeons. Accordingly, the reliability, reproducibility and ergonomic design of the guidance system are crucial not only for patient safety but also for structured surgical training. While robotic stereotactic systems have demonstrated high technical accuracy and feasibility, it remains unclear whether these advantages translate into reduced operator dependency in routine clinical practice. In particular, evidence is lacking on whether robotic assistance enables less experienced surgeons to achieve procedural efficiency, diagnostic performance, and safety comparable to those of experienced neurosurgeons.

Therefore, the primary aim of this study was to compare the robotic Cirq® platform with the established manual frameless Varioguide® system regarding operative workflow, diagnostic yield, and safety. In addition, we specifically investigated whether robotic assistance reduces experience-related differences between residents and attending neurosurgeons, thereby supporting standardized surgical performance and neurosurgical training.

## Methods

### Patient selection and study design

We conducted a retrospective analysis of consecutive patients with intracerebral lesions of unclear origin, including suspected tumors, abscesses, or inflammatory processes, for whom stereotactic biopsy was indicated between 01/02/2021 and 01/10/2025 in a single high-volume neurosurgical center. Patients were excluded if they had undergone frame-based biopsy, or if essential data was missing from the surgical or histopathological reports. Group assignment was based on the biopsy system that was used: either the MFS or the RA, both integrated into the navigation platform. The unit of analysis was the individual stereotactic biopsy procedure rather than the individual patient, as all primary and secondary endpoints were procedure specific. Initially, as part of a 1:1 approach, 50 RA cases and 50 MFS cases were included in the analysis. To establish temporal comparability (e.g., team composition, perioperative standards), the immediately preceding MFS cases were selected. However, one RA case was excluded due to missing data. To maintain equal group sizes according to the 1:1 approach without introducing a selection bias, the chronologically furthest MFS case was excluded. This resulted in a final cohort of 49 procedures in each group. The validity of chronological matching was assessed by comparing baseline characteristics of both cohorts, including patient demographics (age, gender) and lesion characteristics. To investigate the influence of surgical experience, surgeons were categorized as residents (postgraduate years two to six) or attendings (board-certified neurosurgeons). Prior experience with frameless stereotactic biopsy generally increased with the level of training. As the Cirq® system had only recently been introduced at our institution, no participating surgeon had previous clinical experience with this robotic platform before its implementation. Procedures performed by residents were always conducted under direct supervision of an attending. The data was accessed for research purposes between 01/01/2024 and 01/11/2025.

### Surgical technique

All procedures were performed under general anesthesia in a standardized operative setting. The patient’s head was fixated in a Mayfield three-pin skull clamp and neuronavigation registration was performed using a 3D surface model based on preoperative MRI.

Trajectory planning was conducted preoperatively using contrast-enhanced MRI and performed within the Brainlab planning software (Trajectory 2.5.1.5, Brainlab AG, Munich, Germany). Planning included axial, sagittal, coronal, 3D, and in-sight reconstructions. For lesions located in or near eloquent areas, navigated transcranial magnetic stimulation (nTMS) and diffusion tensor imaging fiber tracking (DTI FT) data were registered and integrated into the platform. These datasets were visualized during trajectory planning to optimize accuracy and minimize the risk of functional damage. The target point within the lesion was selected based on lesion contouring.

In the MFS group, the preplanned trajectory was manually aligned intraoperatively using the mechanical stereotactic arm with adjustable joints.

In the RA group, the Cirq® robotic alignment system was mounted to the operating table after patient positioning and head fixation. Following sterile draping, the alignment module was manually positioned close to the planned entry point. The preplanned trajectory was then selected at the navigation workstation, after which the system automatically aligned the instrument holder to the predefined trajectory. Once alignment was completed, the device provided passive mechanical stabilization throughout the procedure. Burr-hole drilling and subsequent biopsy needle insertion were performed manually by the operating surgeon under continuous real-time neuronavigation control. At least three samples were collected from both the core and the periphery of the lesion. The skin was closed using one or two sutures in the standard way.

### Outcome parameters

The primary outcome was defined as the comparison of safety, surgical time and diagnostic yield for both the RA and MFS cohorts after stratification by surgeon experience level. The diagnostic yield was defined as the proportion of procedures that resulted in a conclusive histopathological diagnosis.

Surgical times were determined by total operative time (defined as the entire duration from the initial entry into the operating room to the final exit) and predefined workflow intervals to ensure reproducibility: entry to end of positioning (duration from room entry until the end of patient positioning, patient positioning was completed, parallel to the induction of general anesthesia, preoperative preparations, the spatial integration and unsterile setup of the respective system in use, the review of preoperative planning, and verification of functionality); end of positioning to incision (duration from the end of patient positioning until skin incision, during which neuronavigation registration, sterile draping and trajectory alignment of the respective biopsy system are performed); incision to suture (time from skin incision to the last skin suture).

Safety was evaluated by the incidence of perioperative complications, and diagnostic performance by the rate of conclusive histopathological diagnosis and concordance between preoperative radiological suspicion and final histopathological findings. Complication was defined as any new postoperative neurological deficit, seizure, or decrease in the Glasgow Coma Scale (GCS) as well as postoperative hemorrhage documented during the postoperative hospital stay. Neurological deficits were further classified as temporary or permanent based on their status at hospital discharge.

### Ethical approval

This study was approved by the local institutional review board (Ethical committee of the Technical University of Munich, reference number 200/21 S). Due to the anonymized data handling, the requirement for informed consent was waived. All procedures were conducted in accordance with the Declaration of Helsinki.

### Statistical analysis

All statistical analyses were performed using IBM SPSS Statistics (version 29.0.2.0; IBM Co., Armonk, NY, USA) and GraphPad Prism (version 10.2.1; GraphPad Software, La Jolla, CA, USA). Descriptive statistics are presented as mean ± standard deviation (SD) or as median and range, as appropriate. Assumptions of normality were evaluated using the Shapiro-Wilk test. Continuous variables were compared using an unpaired t-test or a Mann–Whitney U test depending on data distribution. Categorical variables were analyzed using Fisher’s exact test. For operative time and workflow intervals, we used generalized linear models including system type (RA vs MFS), surgeon experience (resident vs attending), and their interaction term (system × experience) to assess whether the effect of experience differed between systems. For positively skewed distributions, a Gamma distribution with a log-link function was specified, and variables with log-normal distributions were transformed using the natural logarithm (ln) for normalization. Statistical significance was defined as p < 0.05.

## Results

### Patient cohort

During the study period, 91 patients underwent a total of 98 frameless stereotactic brain biopsy procedures. Following application of the inclusion and exclusion criteria, 98 procedures (49 RA and 49 MFS) were included in the final analysis. Seven patients underwent more than one biopsy procedure for separate clinical indications.

The mean age was 61 years (range: 19–89; SD = 15.7) in the RA group and 59 years (range: 19–91; SD = 18.4) in the MFS group (p = 0.57). The gender distribution was comparable between groups, with 59.2% male patients in the RA group and 61.2% in the MFS group (p = > 0.99). All patient demographics are displayed in Table 1.

**Table 1:** Patient demographics.

	MFS (n=49)	RA (n=49)	p
Age (mean ± SD, range)	59 ± 18.4, 19 – 91	61 ± 15.7, 19 – 89	0.57
Sex (n)			>0.99
• Male (n=54)	30	29	
• Female (n=37)	19	20	
Karnofsky Performance Scale, preoperative (median, range)*	80 (40 – 100)	80 (50-100)	>0.99
Preoperative PET-Scan (n=26)	13	13	>0.99
Preoperative nTMS with DTI (n=17)	12	5	0.11
Experience level			0.73
• Resident-performed surgery (MFS n=14, RA n=16)	30	34	
• Attending-performed surgery (MFS n=5, RA n=4)	19	15	

*out of 40 patients with an oncological diagnosis in the MFS group and 42 patients with an oncological diagnosis in the RA group.

### Surgical data and lesion characteristics

Preoperative nTMS, including DTI fiber tracking of motor and/or language pathways, was performed in 12 cases in the MFS group and five cases in the RA group. At our institution, DTI FT is routinely acquired only as part of the nTMS workflow; therefore, the number of patients undergoing DTI FT was identical to the number undergoing nTMS.

PET imaging was available in 13 patients in each group. Image fusion and multimodal data integration were feasible in all cases. Multimodal data integration and image fusion were successfully achieved in all cases. Most lesions were located in the frontal lobe with 39.3% in the RA group and 43.1% in the MFS group (see Table 2).

**Table 2:** Data on tumor characteristics, histopathological findings, and surgery-related data.

	MFS (n=49)	RA (n=49)	p
Hemisphere			>0.99
• Right	24	24	
• Central	3	3	
• Left	22	22	
Tumor location			0.34
• Frontal	22	18	
• Temporal	6	6	
• Parietal	6	5	
• Occipital	0	3	
• Basal ganglia	1	1	
• Insular	0	4	
• Thalamic	6	7	
• Cerebellar	2	0	
• Corpus callosum	3	3	
• Other	3	2	
Histopathological findings			0.86
• Glioblastoma, IDH-wildtype, CNS WHO grade 4	17	15	
• Abscess	5	11	
• Lymphoma	10	5	
• Inflammatory lesion, non-infectious	3	5	
• No pathologic cells / reactive tissue	4	4	
• Astrocytoma, IDH-mutant, CNS WHO grade 3 and 4	2	2	
• Metastasis	1	1	
• Low-grade glioma	1	1	
• Past ischemia	0	1	
• HGNET	1	0	
• Multiple sclerosis	1	0	
• ADEM	1	0	
• Neurocytoma	0	1	
• Necrotic tissue	1	1	
• Hematoma	2	2	
Diagnosis secured			>0.99
• Yes	45	45	
• No	4	4	
Duration of surgery (min)			
• Entry to end of positioning (median (IQR))	13.0 (10.5 - 26.5)	31.0 (15.0 - 50.0)	**<0.0001**
• End of positioning to incision (median (IQR))	30.0 (25.0 - 41.0)	34.0 (24.0 - 44.0)	0.53
• Incision to suture	19.67 ± 11.62	21.65 ± 12.57	0.34
• Total OR time	97.98 ± 27.21	115.6 ± 34.07	**0.0036**
Total adverse events	12	9	0.62
• Postoperative bleeding	5	6	>0.99
◦ symptomatic	4	6	
◦ asymptomatic	1	0	
• New neurological deterioration (any)	11	9	
◦ temporary	7	4	
◦ persistent	4	5	
◦ of these, associated with postoperative bleeding	4	6	

Note: Adverse event categories are not mutually exclusive. Therefore, they are not additive: a single procedure may be counted under both postoperative bleeding and new neurological deterioration. Total adverse events refers to the number of procedures with at least one adverse event.

Lesion eloquence was comparable between the groups (MFS: 36/49 [73.5%] vs. RA: 32/49 [65.3%], p = 0.511). Likewise, the mean biopsy trajectory length did not differ significantly (MFS 3.80 ± 1.61 cm vs. RA 3.78 ± 1.52 cm, p = 0.949).

In three patients with two distinct lesions (two in the RA group and one in the MFS group), both targets were biopsied within a single surgical session. These cases were retained in the analysis, as their operative times fell within the range of the respective group averages, and a sensitivity analysis with and without these cases revealed no relevant impact on the results.

The histopathological findings are displayed in Fig 1.

**Fig 1:**
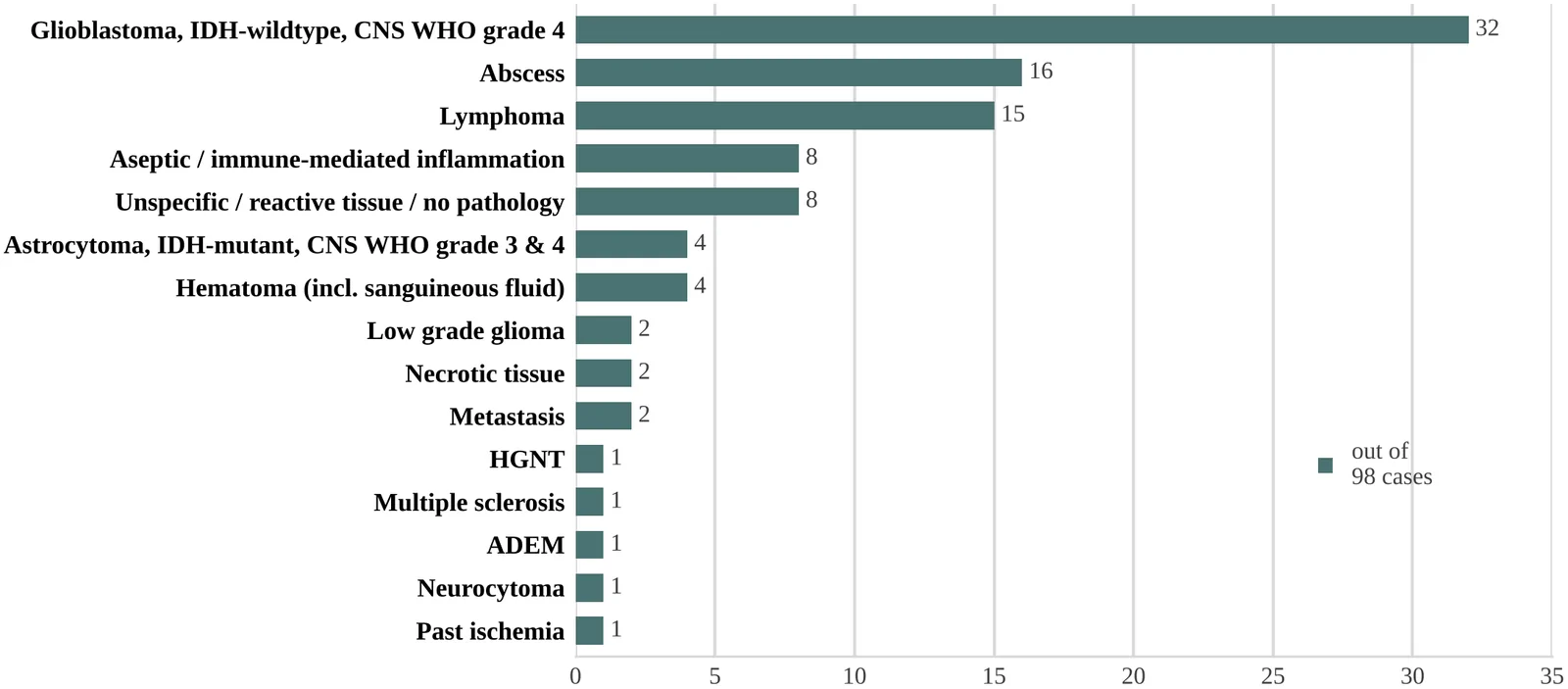
Distribution of histopathological diagnosis for conclusive biopsies in both groups.

### Overall comparison between the robotic and manual system

Across the entire cohort, mean total operative time (entry to exit) was longer in the RA group (115.6 ± 34.1 min) compared to the MFS group (97.9 ± 27.2 min; 95% CI [5.3, 30.0], Cohen’s d = 0.60, p = 0.0036). The entry to end of positioning interval was significantly longer with RA (median 31.0 min, IQR 15.0–50.0 min vs. median 13.0 min, IQR 10.5–26.5 min; 95% CI [5.0, 21.0], r = 0.40, p < 0.0001, see Fig 2), whereas end of positioning to incision (median 34.0 min, IQR 24.0–44.0 min vs. median 30.0 min, IQR 25.0–41.0 min; 95% CI [−3.0, 6.0], r = 0.06, p = 0.53) and incision to suture times (21.7 ± 12.6 min vs. 19.7 ± 11.6 min, 95% CI [−2.9, 6.8], Cohen’s d = 0.19, p = 0.34) did not differ significantly. Conclusive histopathological diagnoses were achieved in 91.8% of cases in both groups (p > 0.99), with diagnostic concordance between radiological and histological findings in 81.4% of all cases (p = 0.79). A sensitivity analysis of the results excluding repeated biopsies was performed to account for possible intra-patient correlation. This did not alter the statistical significance or direction of the results, confirming the robustness of the primary outcomes.

**Fig 2:**
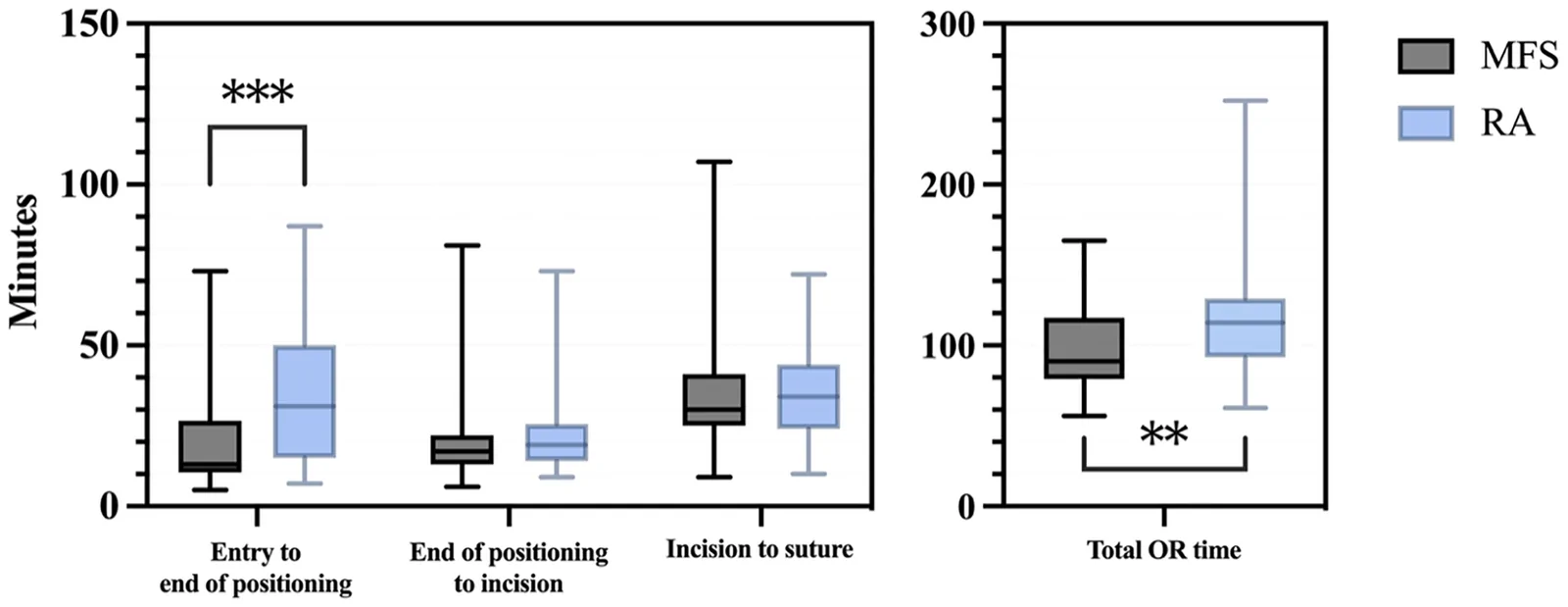
Boxplots showing OR times for entry to end of positioning, end of positioning to incision, incision to suture and total OR time from entry to exit of RA vs. MFS.

In the RA group, rapid histopathological assessment of fresh tumor tissue was performed intraoperatively in 63.3% of procedures, while 36.7% were submitted for definitive histopathological examination only. A second intraoperative rapid section was required in 10.2% of cases due to inconclusive initial findings. This yielded results concordant with the final diagnosis in 60% of cases.

In the MFS group, rapid histopathological assessment was performed in 69.4% of cases. A second rapid section was obtained in 6.1% of cases, leading to a definitive diagnosis in one case (33.3%). In the remaining two cases, a third rapid section remained non-specific, and definitive histopathology ultimately provided a conclusive diagnosis in both cases.

Overall, perioperative complications occurred in 21 patients (21.4% of all procedures), including nine in the RA group (18.4%) and 12 in the MFS group (24.5%; p = 0.62). Postoperative hemorrhage was detected in 11 patients (11.2%), comprising six cases in the RA group and five in the MFS group (p > 0.99). Ten of these were symptomatic, whereas one represented an incidental radiological finding without clinical relevance. New neurological deterioration, including deterioration related to symptomatic hemorrhage, occurred in 11 MFS and nine RA procedures. In the MFS group, seven deteriorations (14.3%) were temporary and four (8.2%) persisted until discharge. In the RA group, four (8.2%) were temporary and five (10.2%) persisted until discharge. In two patients with persistent deficits, one in each group, neurological deterioration was considered at least partly attributable to progression of the underlying disease. No postoperative infections occurred (see Table 2 and Fig 3).

**Fig 3:**
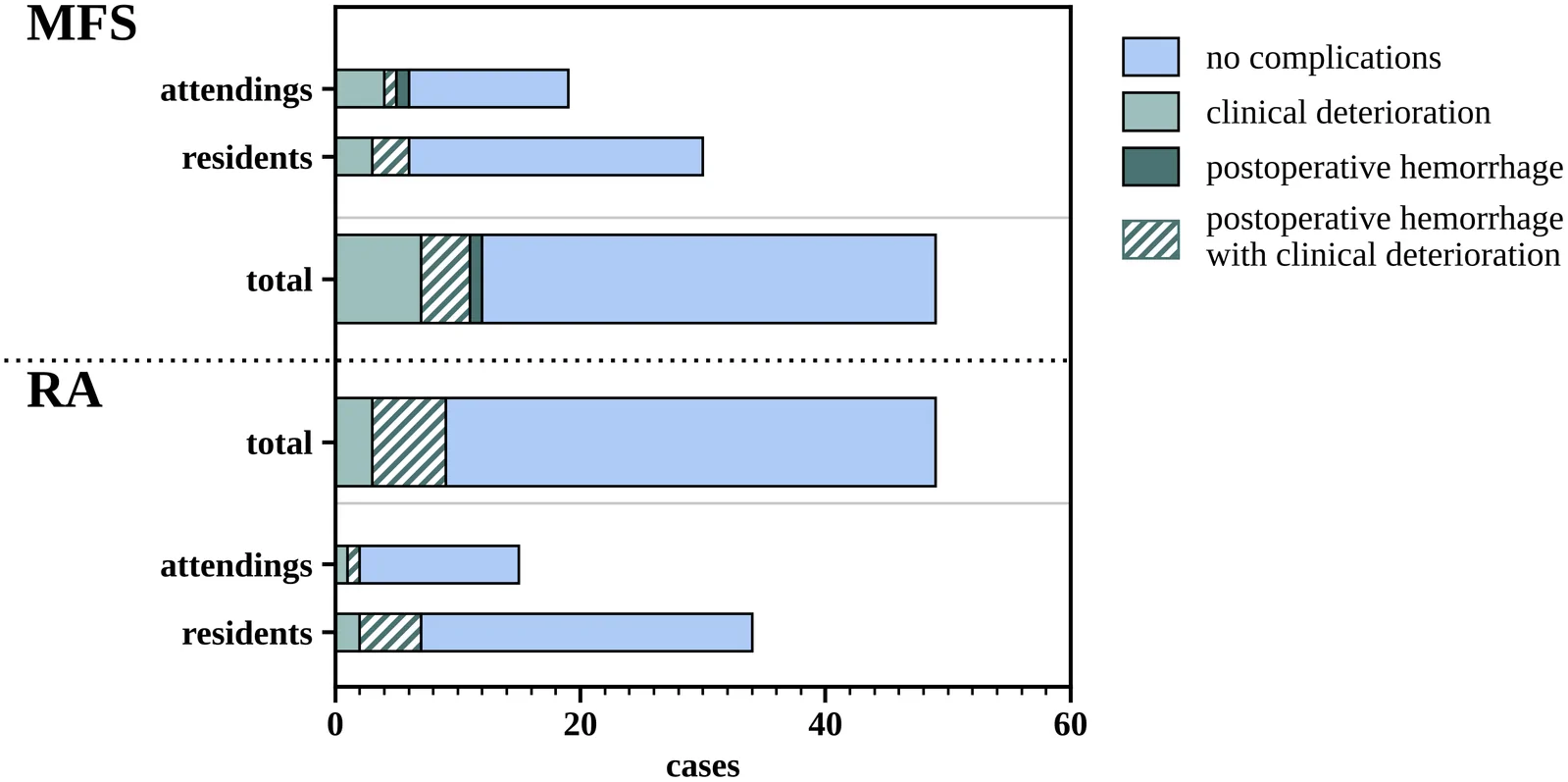
Complication rates for the MFS (Varioguide®) and the RA (Cirq®).

### Influence of surgeon experience on operative efficiency, diagnostic yield and safety

When stratified by surgeon experience, variability in operative performance among residents was lower in the RA group compared with the MFS. The coefficient of variation for total OR time among residents decreased from 28.1% with MFS to 23.3% with RA, indicating a more standardized procedural workflow under robotic guidance. A similar pattern was observed for incision to suture time, with variability decreasing from 69.0% to 34.9% when using RA.

In the RA group, residents achieved shorter mean total OR times compared with attending surgeons (108.2 ± 25.2 minutes vs. 132.3 ± 45.3 minutes; 95% CI [3.8, 44.4], Δ = –24.1 minutes, Cohen’s d = 0.74, p = 0.0208). In contrast, within the MFS group, residents had longer mean total OR times than attendings (Δ = +10.7 minutes), a difference that did not reach statistical significance (95% CI [−5.2, 26.6], Cohen’s d = 0.40, p = 0.1832).

For incision to suture time, residents in the RA group again demonstrated superior efficiency, with a mean duration of 17.97 ± 6.3 minutes compared with 30.0 ± 18.5 minutes among attendings (95% CI [4.9, 19.1], Δ = –12.0 minutes, Cohen’s d = 0.93, p = 0.0043). Conversely, within the MFS group, only a negligible difference was observed between residents and attendings (95% CI [−7.3, 6.6], Δ = –0.36 minutes, Cohen’s d = 0.18, p = 0.5335). Overall, the difference in mean total OR time between residents and attendings was –24.1 minutes in favor of residents within the RA cohort versus +10.7 minutes within the MFS cohort. Correspondingly, incision to suture time differed by –12.0 minutes with RA compared with –0.36 minutes with MFS, suggesting that robotic assistance can reduce experience-dependent differences in operative efficiency across key workflow intervals (see Fig 4). The multivariable analysis complemented these results: *system × experience* interaction terms were significant for end of positioning to incision (p = 0.030), incision to suture (p = 0.018), and total OR time (p = 0.003), but not for entry to end of positioning (p = 0.784). This supports the hypothesis that the effect of robotic assistance on operative time could depend on surgeon experience. While attendings required longer incision to suture times with RA (p = 0.031), whereas residents were significantly faster with Cirq® in the neuronavigation and alignment related interval end of positioning to incision (p = 0.019).

**Fig 4:**
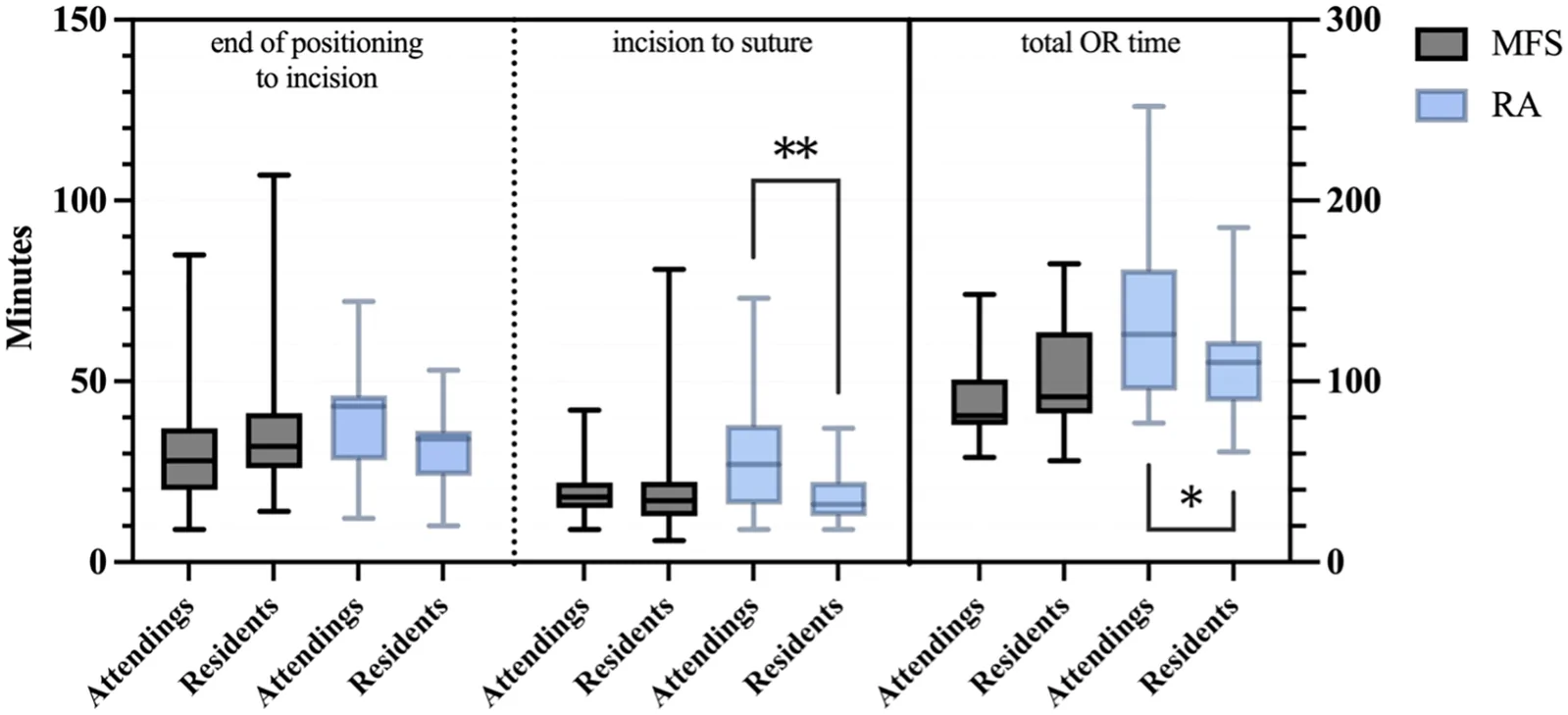
Boxplots showing OR times for end of positioning to incision, incision to suture and total OR time for the MFS (Varioguide®) and the RA (Cirq®), divided by the experience level of the surgeons.

Diagnostic yield was 91.8% in both groups (p > 0.99). After stratification by surgeon experience, diagnostic yield remained high, with conclusive histopathological diagnosis made in 33 out of 34 (97,1%) procedures performed by residents and 12 out of 15 (80,0%) procedures performed by attendings in the RA cohort, compared with 29 out of 30 (96,7%) and 16 out of 19 (84,2%) in the corresponding MFS subgroups (Table 3).

**Table 3:** Diagnostic yield and complication rates by biopsy system and surgeon experience.

System	Surgeon level	n	Conclusive diagnosis	Complications
MFS	Resident	30	29 (96.7%)	6 (20.0%)
MFS	Attending	19	16 (84.2%)	6 (31.6%)
RA	Resident	34	33 (97.1%)	7 (20.6%)
RA	Attending	15	12 (80.0%)	2 (13.3%)

Note: Percentages refer to the proportion of procedures within each subgroup. Diagnostic yield did not differ significantly between residents and attendings in either the RA cohort (p = 0.08) or the MFS cohort (p = 0.29). Complication rates did not differ significantly between residents and attendings within either system, or between systems within either experience level (all p > 0.25).

Complication rates were comparable between residents and attendings within each subgroup. Multivariable logistic regression confirmed that neither biopsy technology (p = 0.95), nor experience level (p = 0.36) significantly affected complication rates (Table 4).

**Table 4:** Operative times by biopsy system and surgeon experience.

	MFS residents (n = 30)	MFS attendings (n = 19)	p	RA residents (n = 34)	RA attendings (n = 15)	p
Entry to end of positioning (IQR)	16.0 (11.0 - 27.5)	11.0 (7.0 - 17.0)	0.0211	33.5 (16.5–52.75)	26.0 (14.0 - 49.0)	0.5087
End of positioning to incision	36.23 ± 17.16	31.37 ± 17.0	0.1462	31.79 ± 10.16	38.53 ± 14.65	0.0689
Incision to suture	19.53 ± 13.48	19.89 ± 8.21	0.5335	17.97 ± 6.26	30.0 ± 18.48	0.0043
Total OR time	102.1 ± 28.73	91.4 ± 23.88	0.1832	108.2 ± 25.22	132.3 ± 45.26	0.0208
CV total OR time	28.13%	26.12%		23.30%	34.20%	

## Discussion

This study provides a comparative analysis of two frameless stereotactic systems for intracerebral biopsy, focusing on the impact of surgeon experience on procedural performance. In our cohort, residents using RA achieved equal or shorter total OR times compared with attendings, while remaining non-inferior regarding diagnostic yield and complications. This pattern was not observed with the manual system, suggesting that robotic guidance can reduce the effect of limited experience on operative efficiency without compromising safety.

The ability of residents to perform procedures as efficiently as attendings when assisted by a robotic platform highlights an important advantage of the RA. These findings support the concept of RA as a standardizing tool in an academic environment: by automating trajectory alignment and stabilizing the biopsy path, the system may reduce the number of experience-dependent manual steps, thereby narrowing the performance gap between junior and senior surgeons. This is in line with reports from other surgical disciplines, where robotic assistance has been shown to facilitate early acquisition of technical skills and to improve performance consistency across different levels of training. Ultimately, such systems may not only support surgical education but also enhance patient safety by ensuring reliable operative quality independent of surgeon experience. However, this finding should be interpreted with caution. Case allocation was not randomized and differences in lesion complexity, procedural characteristics, or intraoperative task allocation between residents and attendings may have contributed to the observed differences in operative time. Although no systematic disparities were identified, potential confounders cannot be excluded in this retrospective study design.

Importantly, the observed reduction in experience-related differences should not be interpreted as suggesting that robotic assistance replaces surgical expertise. Successful stereotactic biopsy fundamentally depends on appropriate patient selection, precise trajectory planning and detailed knowledge of neurovascular anatomy and eloquent brain structures [12]. The robotic system assists in the execution and stabilization of a predefined trajectory but does not influence the underlying surgical decision-making process. This is supported by previous large prospective series demonstrating improved procedural reproducibility and accuracy of robotic stereotactic systems while emphasizing that the advantages relate primarily to technical execution rather than surgical decision-making [13].

Several prior studies have shown that stereotactic biopsy in general remains a reliable and safe method for establishing a histopathological diagnosis, especially for deep-seated or radiologically ambiguous lesions. The Varioguide® system has been widely used as a frameless alternative due to its flexibility and integration into navigation systems [3,4]. However, it remains a manually controlled mechanical system, which requires the surgeon to perform all alignments.

Other, more recent studies have reported that robotic systems improve targeting precision and intraoperative reproducibility in stereotactic procedures [8,9]. These advantages were recently confirmed in a pediatric case series by van Baarsen et al., in which the Cirq® system proved to be both feasible and safe for brain tumor biopsies [14]. Our findings can further support this.

The longer setup times should be interpreted in the context of an institutional learning curve. Because procedures were distributed among multiple surgeons within a relatively small cohort, modeling an individual learning curve was not feasible. However, whereas MFS has been the standard technique in our department for more than a decade, the RA platform was newly introduced during the study period. It is therefore plausible that setup times for RA-assisted biopsies will decrease as institutional familiarity and routine use increase.

Despite requiring longer setup times, we hypothesize that robotic systems may facilitate easier and more straightforward handling of the operative steps once aligned. This procedural efficiency may be attributed to the inherent mechanical stability of the robotic arm, eliminating the need for manual readjustment. Additionally, the automatic alignment of the pre-planned trajectory minimizes small deviations and enables more precise execution of the biopsy. Our analysis revealed that residents achieved equal or shorter procedure times than attendings when using the RA, particularly in the incision to suture interval and total operative time. This suggests that robotic assistance may reduce the influence of operator experience. In contrast, the MFS showed no difference between experienced and less experienced surgeons, reflecting its dependence on manual trajectory alignment and intraoperative adjustments.

Regarding safety, complication rates did not differ significantly between the two systems. Postoperative hemorrhages occurred in 11 procedures, ten of which were symptomatic. However, none required surgical intervention. Within both systems, the diagnostic yield and complication rates did not differ significantly between residents and attendings. These findings suggest that the robotic arm allows less experienced surgeons to achieve outcomes comparable to senior neurosurgeons, without an increase in complication rates. Conclusively, our results confirm the reliability of frameless biopsy techniques and support the integration of robotic tools without compromising diagnostic accuracy. These findings are in line with recent comparative studies demonstrating that robot-assisted stereotactic biopsy provides diagnostic yield and safety comparable to established stereotactic techniques while maintaining high procedural accuracy [15].

### Limitations

Our study was subject to several well-known limitations due to its retrospective, observational, explorative and single-center design.

First, the number of RA cases in our cohort was relatively small due to the system’s recent introduction. In contrast, the MFS has been well established in our department for over a decade, which likely contributed to faster setup and shorter total procedure times in that group. The observed differences in workflow duration may therefore, at least in part, reflect an inherent learning curve associated with the adoption of a new robotic system**,** rather than procedural inefficiency. Regarding data analyses and case complexity, another limitation must be added: Although lesion eloquence and biopsy trajectory length were comparable between groups, other aspects of case complexity, such as lesion size or imaging characteristics, were not formally incorporated into the statistical analysis and may have acted as residual confounders. Furthermore, objective measures of stereotactic accuracy, such as entry point deviation, target error or trajectory deviation, were not recorded and therefore could not be analyzed retrospectively. Future studies should incorporate these parameters to further evaluate the potential technical advantages of robotic assistance.

Second, although all procedures were supervised or performed by board-certified neurosurgeons, the operative teams included surgeons at various stages of training. This heterogeneity could have introduced variability in handling and timing. However, this factor was consistent across both groups and is representative of a typical academic neurosurgical environment. In addition, procedure numbers for individual surgeons were not analyzed separately, and prior experience with frameless stereotactic biopsy likely varied accordingly. Altogether, the study reflects supervised team performance rather than resident-independent surgery.

Third, the multivariable analysis should be interpreted with caution, given that the relatively small number of adverse events limited the statistical power to detect independent predictors of complications. Consequently, the regression analyses should be considered exploratory. It would require confirmation in larger cohorts.

Fourth, potential selection bias cannot be excluded, as the choice of system was not randomized and may have been influenced by operating room availability or surgeon preference. In addition, temporal bias cannot be entirely excluded, as the RA and MFS procedures were performed during partially different time periods. Although the MFS cohort was selected from cases immediately preceding the introduction of the RA system to minimize differences in the workflows and perioperative standards, the observed differences in operative duration may still reflect temporal changes. Finally, the relatively limited sample size, particularly for subgroup and multivariable analyses, may have reduced statistical power. The results should therefore be interpreted with appropriate caution until confirmed in larger prospective studies.

## Conclusions

The present study demonstrates that robotic-assisted frameless biopsy using the Cirq® system is safe, feasible, and effective. Although total operative times were longer during the initial phase of implementation, this primarily reflected setup and calibration efforts rather than procedural inefficiency. Comparable diagnostic yield, safety, and operative performance across different levels of surgical experience suggest that robotic guidance may support procedural standardization in clinical practice. Future prospective studies are warranted to determine whether these findings translate into sustained improvements in operative efficiency, learning curves, and neurosurgical training.
